# P-1748. Rethinking Ocular Screening in Candidaemia: A Real-World Analysis to Inform National Policy Harmonization

**DOI:** 10.1093/ofid/ofaf695.1919

**Published:** 2026-01-11

**Authors:** Sajeevan Rasanantham, Heather Dolby, Rory Houlihan

**Affiliations:** South Tees NHS Foundation trust, Middlesbrough, England, United Kingdom; James Cook University Hospital, Middlesbrough, England, United Kingdom; James Cook University Hospital, Middlesbrough, England, United Kingdom

## Abstract

**Background:**

Ocular involvement in candidaemia, while rare, carries the risk of devastating visual sequelae. Despite this, guideline recommendations remain inconsistent-ranging from universal screening to selective, symptom-driven assessment. The 2025 ECMM Global guideline now advocates a risk-stratified, patient-centered approach rather than blanket fundoscopy. In light of this evolution, we assessed real-world screening practices in a UK tertiary centre to determine how well current practices align with emerging consensus and whether they may guide future national recommendations.

**Figure 1:**
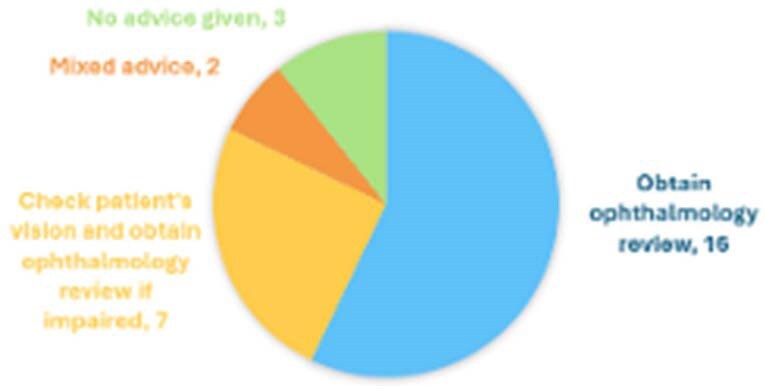
Advice to ward clinicians from microbiology specialists regarding assessment for ocular candidiasis, in the audit cohort (n=28) (Advice, Number of patients).

**Methods:**

We conducted a retrospective observational study of all adult patients( ≥ 18 years) with blood culture confirmed candidaemia between June 1,2022, and June 1,2023. Data included opthalmology referral status, timing, microbiology consultation advice, and fundoscopy findings. Patients were stratified by clinical risk factors, care setting, and microbiologist input.

**Results:**

Of 28 patients identified, only 7(25%) underwent opthalmology review with dialated fundoscopy; 5 within seven days, all in critical care. Among the 21 not reviewed, 28.6% had rejected referrals, while 42.9% lacked any referral attempt. Microbiologist documented ocular screening recommendations in 89.3% of cases within three days: 64% advised universal review, 28% endosed symptom-based referral, and 8% gave mixed advice. No cases of ocular candidiasis were diagnosed. Importantly, no visual symptoms were reported, and all the patients had responded clinically to antifungal therapy. Our data suggest both low pre-test probability and significant heterogeneity in practice, potentially influenced by ambiguous or outdated guidelines.

**Conclusion:**

In this cohort, the absence of ocular disease and the variability in screening reflect the clinical equipoise underpinning current practice. Rather than routine fundoscopy for all, risk-adopted pathways; incorporating symptoms, clinical course, and care setting; may ensure diagnostic stewardship without unnecessary resource use. This study provides real world evidence to support a paradigm shift toward evidence-aligned, equity focused, and operationally feasible screening strategies for ocular candidiasis.

**Disclosures:**

All Authors: No reported disclosures

